# Super-resolution reconstruction of industrial PET images using a prior-knowledge-based generative adversarial network

**DOI:** 10.1038/s41598-025-33267-1

**Published:** 2026-01-06

**Authors:** Mingwei Zhu, Min Zhao, Min Yao

**Affiliations:** 1 School of Mechanical Engineering, NanTong University, Nantong, 226019 Jiangsu China; 2 College of Automation Engineering, Nanjing University of Aeronautics and Astronautics, Nanjing, 211106 China

**Keywords:** Super-resolution reconstruction, Residual block, Positron emission tomography, Generative adversarial networks, Non-destructive testing, Engineering, Mathematics and computing

## Abstract

Positron tomography technology (PET) can adapt to complex on-site environments, enabling industrial non-destructive testing without disturbance or damage. PET super-resolution reconstruction aims to reduce detection costs and improve accuracy, making it highly valuable for research. In this study, we propose a generative adversarial network (GAN)-based super-resolution model for industrial PET images that incorporates prior knowledge to address issues such as detail loss and artifact distortion in existing algorithms. We design a texture enhancement network to extract detailed features and employ a connection network to fuse texture and super-resolution features, enhancing texture details. Additionally, we introduce texture loss and super-resolution loss to further improve the model’s performance. Experimental results demonstrate that the proposed method enhances super-resolution image quality in both visual and objective evaluation metrics and has been validated in practical industrial detection.

## Introduction

Positron imaging technology can achieve visual defect imaging of enclosed industrial parts cavities. Compared with commonly used methods such as industrial CT^1^, ultrasonic testing^2^, and magnetic particle testing^3^, this technology is not affected by complex environments, and the detection process is safer, more applicable to a wide range of scenarios, and more efficient. At the same time, it does not have a secondary impact on the detection object. However, due to hardware limitations and restricted acquisition time, industrial PET images often suffer from blurred edges, loss of structural details, and insufficient clarity, making super-resolution reconstruction crucial for enhancing image quality in practical applications.

Although PET (Positron Emission Tomography) is traditionally used in biomedical imaging, recent research has extended its application into industrial PET, enabling nondestructive internal visualization of enclosed metallic or composite structures. This indicates that PET has the potential to address scenarios in which conventional imaging modalities such as X-ray CT and ultrasound encounter limitations caused by dense materials or complex geometries. Several studies have explored PET’s industrial potential in areas such as flow field visualization, defect detection in composite materials, and transport process analysis within porous media^4–6^. These developments highlight PET’s ability to provide high-resolution internal information that is otherwise inaccessible and underscore the need for enhanced reconstruction methods. Motivated by these challenges, our work aims to improve the reconstruction quality of industrial PET images and support the practical advancement of this emerging imaging technology.

The classical PET super-resolution reconstruction algorithms can be classified into interpolation-based methods, reconstruction-based methods, and learning-based methods. Interpolation-based methods^7–9^ are easy to implement, computationally simple, and relatively fast. However, these algorithms cannot leverage high-level semantic information in the image, and may result in blurring, unclear textures, and jagged edges on complex images, leading to artifacts. Reconstruction-based method^10–12^ can improve the aforementioned issues, but the reconstructed images tend to be overly smooth, and the computational cost is high with low efficiency.

In recent years, learning based methods, especially deep learning methods, have made significant progress in the field of image super-resolution. The basic idea is to automatically learn the optimal down-sampling method by training a large amount of sample data. The currently widely used methods include those based on convolutional neural networks^13^, generative adversarial networks (GAN)^14^, and encoding decoding^15^. Dong et al.^16^ first used convolutional neural networks for image super-resolution reconstruction in 2015. By directly learning the end-to-end mapping relationship between high/low resolution images, they constructed a super-resolution convolutional network (SRCNN), which improved the super-resolution reconstruction effect of images. Kim et al.^17^ proposed a very deep super-resolution (VSDR) model based on SRCNN, which effectively utilizes contextual information through cascaded small filters, uses residual networks for feature learning, improves the learning efficiency of multi-layer neural networks, shortens training time, and improves the accuracy of reconstructed images.

Ledig et al. proposed SRGAN (Super Resolution using a Generative Adversarial Network)^18^ for super-resolution reconstruction in 2017. This model was the first to apply Generative Adversarial Networks (GANs) to image super-resolution reconstruction. Shang et al.^19^ develop a super resolution network with receptive field block based on Enhanced SRGAN (RFE-ESRGAN), which extracts multi-scale features of images and alternately uses different upsampling methods to reduce computational complexity, achieving perceptual extreme super-resolution reconstruction while maintaining low time complexity while restoring image details and textures.

Heydari et al.^20^ proposed an image super-resolution variational autoencoder model (SRVAE), which uses two sets of encoder decoders to learn the mapping relationship between high-resolution and low-resolution images, thereby achieving image super-resolution reconstruction. Chir et al.^21^ proposed a deep variational autoencoder model that uses transfer learning on a pre trained encoder for image super-resolution reconstruction, and achieved good reconstruction results.

The above methods have achieved good results in super-resolution reconstruction of images. However, due to the lack of effective prior information and expression of domain features in industrial positron imaging, using super-resolution reconstruction to obtain images with precise details and clear contours is still a major research focus of positron imaging technology applied in the industrial field. To address this, we introduce Generative Adversarial Networks (GANs), as their adversarial learning mechanism enables the recovery of fine structural details and realistic textures, which are essential for improving the perceptual quality of reconstructed PET images. Moreover, GAN-based models offer a favorable balance between reconstruction fidelity and computational efficiency, making them suitable for real-time industrial non-destructive testing scenarios.

In this study, we develop an industrial PET image super-resolution model based on generative adversarial networks that integrates prior knowledge, enhances image texture details, and repeatedly leverages image domain features through prior knowledge learning to achieve superior PET image super-resolution results.

Overall, the main contributions are as follows:

- Constructed a texture enhancement network to enhance high-level pixel information, enabling the super-resolution model to better capture and restore fine-grained details in PET images.
- Constructed a fusion network to repeatedly requisition and integrate image features, thereby improving feature utilization efficiency and optimizing overall network performance.
- Improved the loss function by incorporating texture loss and super-resolution loss, which effectively enhances the network’s capabilities in detail reconstruction and super-resolution accuracy.

## Method

Generative Adversarial Networks (GANs) consist of a generator and a discriminator. The fundamental principle involves iteratively updating the network parameters through a zero-sum game between the generator and the discriminator until the model converges to a Nash equilibrium. Ultimately, an effective generator is trained to produce clear, high-quality, and high-resolution images. GANs exhibit strong modeling capabilities, particularly for small sample datasets, and are able to learn representative features even when training data is limited. Therefore, in this study, GAN is adopted as the foundational framework for the super-resolution reconstruction network of industrial PET images.

**Fig. 1:**
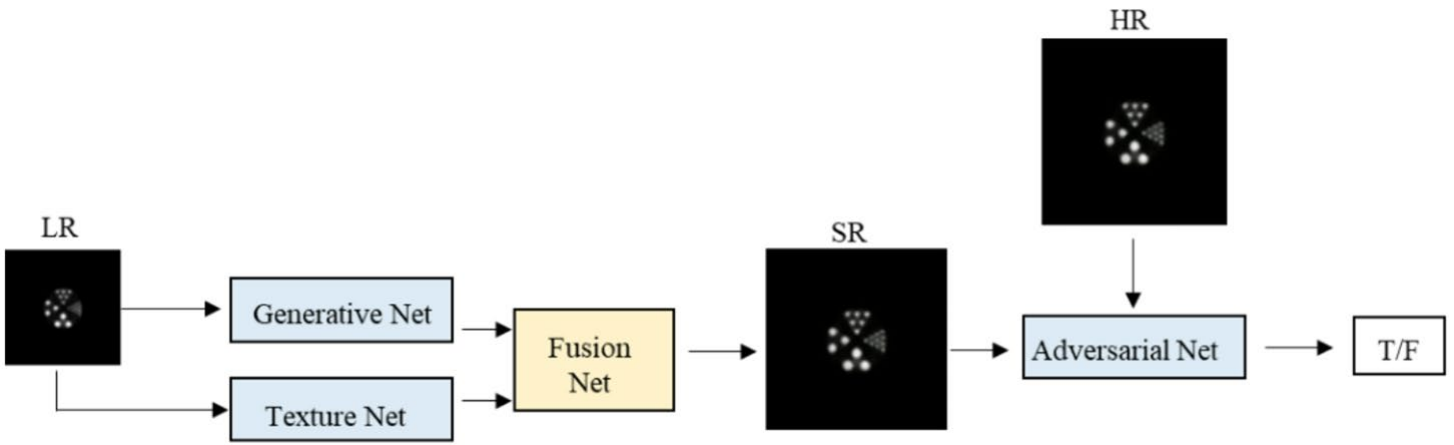
Overall network structure of super-resolution reconstruction model.

Compared to typical natural images, positron emission tomography (PET) images have lower contrast and higher requirements for extracting detailed features. Traditional GANs cannot effectively preserve the detailed features of positron emission tomography images. Therefore, to achieve image super-resolution while preserving the detailed features of the image as much as possible without losing the brightness of the image, this paper proposes a PET image super-resolution reconstruction method based on a generative adversarial network that integrates prior knowledge. The traditional super-resolution network is improved by adding an auxiliary network for the region of interest of the image to extract texture details within the effective area of the image; At the same time, a feature connection network was added to feed back the extracted texture features to the super-resolution network, in order to better characterize the defect features of PET images. The overall network architecture diagram is shown in Fig. 1, and the model consists of three main components: a texture enhancement network, a fusion network for texture and super-resolution features, and a super-resolution reconstruction network.

### Texture network

In practical applications of positron imaging technology, precise localization of defects in the test object is of critical importance. However, many existing methods primarily rely on low-level pixel information and fail to effectively capture high-level semantic features.

**Fig. 2:**
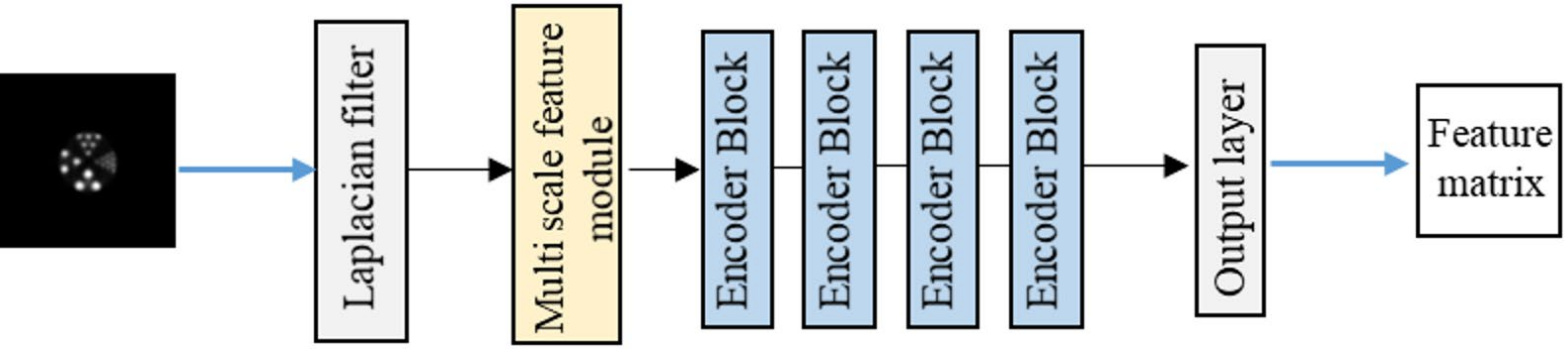
Texture Enhancement Network: a texture feature extraction module, detail enhancement block, texture–super-resolution fusion layer, and defect feature refinement module.

To address this limitation, we designed a texture enhancement network aimed at learning high-level semantic information within the domain of interest in the images, thereby enhancing the reconstruction of high-frequency details. A texture loss function is constructed specifically for industrial PET images, where the learned texture features and high-resolution (HR) images are input into a discriminator for adversarial training. Meanwhile, both low-frequency and high-frequency components of the images are learned. The extracted texture features are then integrated into the super-resolution network via a dedicated attention module. This approach leverages learned prior knowledge to guide the reconstruction process, thereby enabling more effective utilization of high-frequency information. The specific network architecture is illustrated in Fig. 2.

To extract edge and detail features, the Laplacian filter is incorporated into the network for high-frequency feature extraction. Simultaneously, a multi-scale feature extraction module and a self-attention mechanism are introduced. Low-resolution images of size 64 × 64 is fed into the network, and high-frequency features of size 3 × 3 are produced at the output. The activation function used in the convolutional layers is LeakyReLU, with α = 0.2. The multi-scale feature extraction module consists of four parallel convolutional branches and a 1 × 1 convolutional fusion layer. The network performs progressive down-sampling using four convolutional layers with a stride of 2, enabling feature compression and mitigating abrupt loss of image information. The final output layer is a 2 × 2 unpadded convolutional layer.

### Super-resolution network

**Fig. 3:**
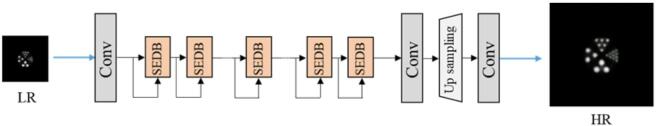
Generative network structure.

**Fig. 4:**
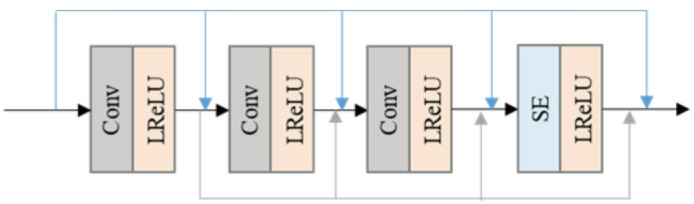
Residual block: each block consists of two convolutional layers and a skip connection.

The PET image super-resolution reconstruction model is based on a generative adversarial network, consisting of an encoding layer, residual layer, up-sampling layer, and output layer. The structure is shown in Fig. 3. Firstly, feature extraction is performed on the input low-resolution image through an encoding layer, which includes a 3 × 3 convolutional layer and a LeakyReLU activation layer, where α = 0.2. The residual block consists of 16 deep residual blocks (PRDB), each containing three 3 × 3 convolutional layers and three LeakyReLU activation layers, the structure is shown in Fig. 4.

The number of residual blocks was empirically determined through ablation experiments to balance model complexity and reconstruction accuracy. Preliminary tests showed that increasing the number of blocks beyond 16 brought negligible performance gains while significantly raising training time and computational cost. Therefore, 16 blocks were adopted as an optimal trade-off between reconstruction fidelity and efficiency.

This setup can extract high-frequency features of images, preserve the output information from the front-end network, and effectively reduce gradient vanishing caused by an increase in network layers. The input image of the network is 64 × 64, which needs to undergo three rounds of 2× up-sampling to obtain a high-resolution image of 512 × 512. The final output layer consists of a 3 × 3 convolutional layer and a Tanh activation layer.

**Fig. 5:**
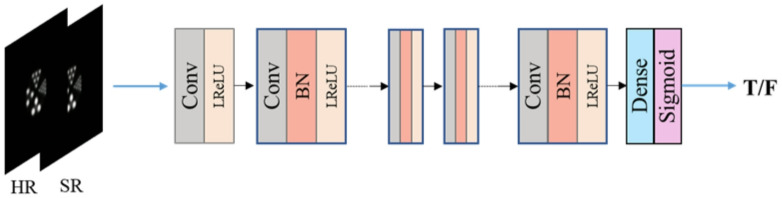
Discriminative network structure.

The discriminator is composed of a fully convolutional network, and the fully convolutional layer can improve the sensitivity of the discriminator network to local details and global texture of the image. The structure is shown in Fig. 5. The network consists of eight layers, with the first six layers including a 3 × 3 convolutional network, the activation layer of the BN layer network using LeakyReLU activation function, the second to last layer including a 3 × 3 convolutional network and sigmoid activation function, and the final layer being the global average pooling layer.

### Fusion network

**Fig. 6:**
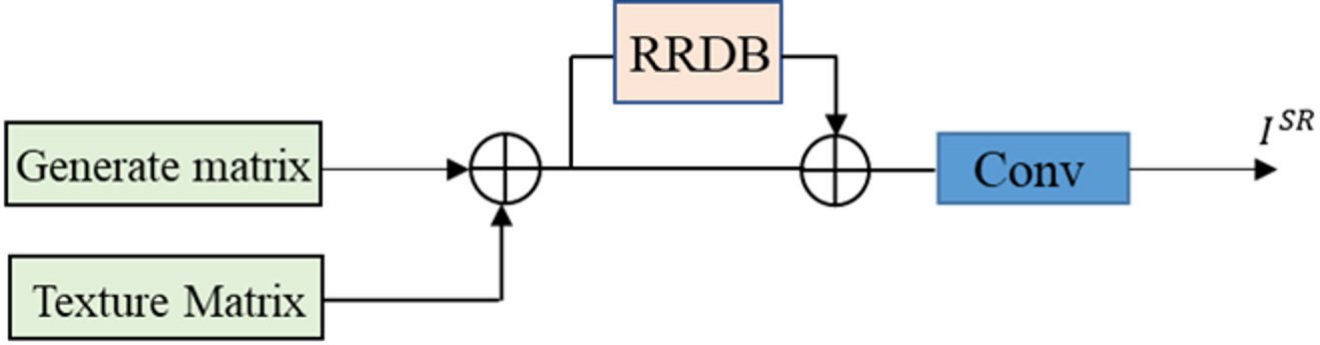
Converged network.

After extracting high-frequency texture features from the image using the texture enhancement network, we incorporate the learned information into the generation network. To achieve this, we constructed a fusion network, as shown in Fig. 6, which integrates the output matrix of the texture enhancement network with the feature matrix generated by the generation network to enhance the quality of image super-resolution reconstruction. The specific implementation method is as follows: the feature matrix obtained from the generation network and the texture matrix obtained from the texture enhancement network are trained using residual learning to achieve weight sharing and repeated feature utilization, thereby preventing the loss of data features. Consequently, texture features are incorporated into the reconstruction network as prior knowledge, facilitating the fusion of low-level image information with high-level semantic information.

### Loss function

To ensure that the generated images in the positron super-resolution reconstruction network conform to the content and structural features of industrial PET images, while preserving the original texture features, a new loss function needs to be constructed to measure the super-resolution performance of the model.

The super-resolution loss was designed to penalize deviations in fine structural details between the generated and ground-truth images. It complements the perceptual and texture losses by directly optimizing pixel-level accuracy, which is critical for preserving the geometric integrity of industrial defect regions.

The loss function of the generative network mainly includes the L1 loss function (Pixel wise Loss), which measures the pixel difference between the generated image and the real image and maintains the similarity of the global structure, as shown in Eq. (1).

1 $$\:{L}_{1}=\frac{1}{N}\sum\:_{i=1}^{N}{\vert \vert {I}_{gen}\left(i\right)-{I}_{real}\left(i\right)\vert \vert }_{1}\:\:\:\:\:\:\:\:\:\:\:\:\:\:\:\:\:\:\:\:\:\:\:\:\:\:\:\:\:\:\:\:\:\:\:\:\:\:\:\:\:\:\:\:\:\:\:\:\:\:\:\:\:\:\:\:\:\:\:\:\:$$

Here, $\:{I}_{gen}$ represents the generated PET image, $\:{I}_{real}$ represents the high-definition real image, and $\:N$ represents the pixel value in the image.

The perceptual loss function utilizes a pre-trained VGG network as a feature extractor to compute the differences between the high-level features of the generated and real images, thereby capturing higher-level semantic information, as shown in Eq. (2).

2 $$\:{L}_{perceptual}=\frac{1}{CHW}\sum\:_{i=1}^{C}\sum\:_{j=1}^{H}\sum\:_{k=1}^{W}{\vert \vert \varphi\:{\left({I}_{gen}\right)}_{i}-\varphi\:{\left({I}_{real}\right)}_{i}\vert \vert }_{2}\:\:\:\:\:\:\:\:\:\:\:\:\:\:\:\:\:\:\:\:\:\:\:\:\:\:\:\:$$

Here, $\:\varphi\:\left(I\right)$ denotes the feature map of image $\:I$ extracted by the VGG network, where $\:C$ is the number of channels, and $\:H\:$and $\:W$ represent the height and width of the image, respectively.

The texture loss function is designed to ensure that the reconstructed image preserves high-frequency structures and edge information similar to those of the original image. It evaluates texture quality by computing the high-frequency features of the image, as shown in Eq. (3).

3 $$\:{L}_{textture}=\frac{1}{N}\sum\:_{i=1}^{N}{\vert \vert G\left({I}_{gen}\left(i\right)\right)-G\left({I}_{real}\left(i\right)\right)\vert \vert }_{1}\:\:\:\:\:\:\:\:\:\:\:\:\:\:\:\:\:\:\:\:\:\:\:\:\:\:\:\:\:\:\:\:\:\:\:\:\:$$

Here, $\:G(\cdot\:)\:$denotes the Laplacian-based multi-scale texture extractor, and $\:N$ is the total number of pixels.

The super-resolution loss function is designed to enhance image resolution while preserving fine structural details, as shown in Eq. (4).

4 $$\:{L}_{SR}=\frac{1}{N}\sum\:_{i=1}^{N}{\vert \vert {I}_{gen}\left(i\right)-{I}_{real}\left(i\right)\vert \vert }_{2}^{2}\:\:\:\:\:\:\:\:\:\:\:\:\:\:\:\:\:\:\:\:\:\:\:\:\:\:\:\:\:\:\:\:\:\:\:\:\:\:\:\:\:\:\:\:\:\:\:\:$$

Furthermore, the overall generator loss function is formulated as a weighted combination of the L1 loss, perceptual loss, texture loss, and super-resolution loss, thereby achieving an optimal balance between global structural accuracy and fine-grained texture realism, as expressed in Eq. (5).

5 $$\:{L}_{gen}={L}_{1}+{\lambda\:}_{1}\cdot \:{L}_{perceptual}+{\lambda\:}_{2}\cdot \:{L}_{tectture}+{\lambda\:}_{3}\cdot \:{L}_{SR}\:\:\:\:\:\:\:\:\:\:\:\:\:\:\:\:\:\:\:\:\:\:\:\:$$

The loss function of the discriminative network is constructed based on the Binary Cross-Entropy loss, as shown in Eq. (6).

6 $$\:{L}_{D}={\mathbb{E}}_{{I}_{real}}\left[log\left(D\left({I}_{real}\right)\right)\right]-{\mathbb{E}}_{{I}_{gen}}\left[log\left(1-D\left({I}_{gen}\right)\right)\right]\:\:\:\:\:\:\:\:\:\:\:\:\:\:\:\:\:\:$$

Here,$\:\:D\left(I\right)$ denotes the predicted score of the input image $\:I$ by the discriminative network.

## Experiments

### Experimental data

The experimental data in this paper was obtained through GATE^22^ (the Geant4 Application for Tomographic Emission) simulation software. We designed and fabricated multiple different types of models, including cavity foreign object models, simple geometric part models, flow field models, and industrial part models, to enrich the diversity of experimental data. The models were sent to GATE, and the OSEM algorithm was used to reconstruct the sampled data, obtaining a high-resolution image of $\:512\:\mathrm{*}\:512$.

**Fig. 7:**
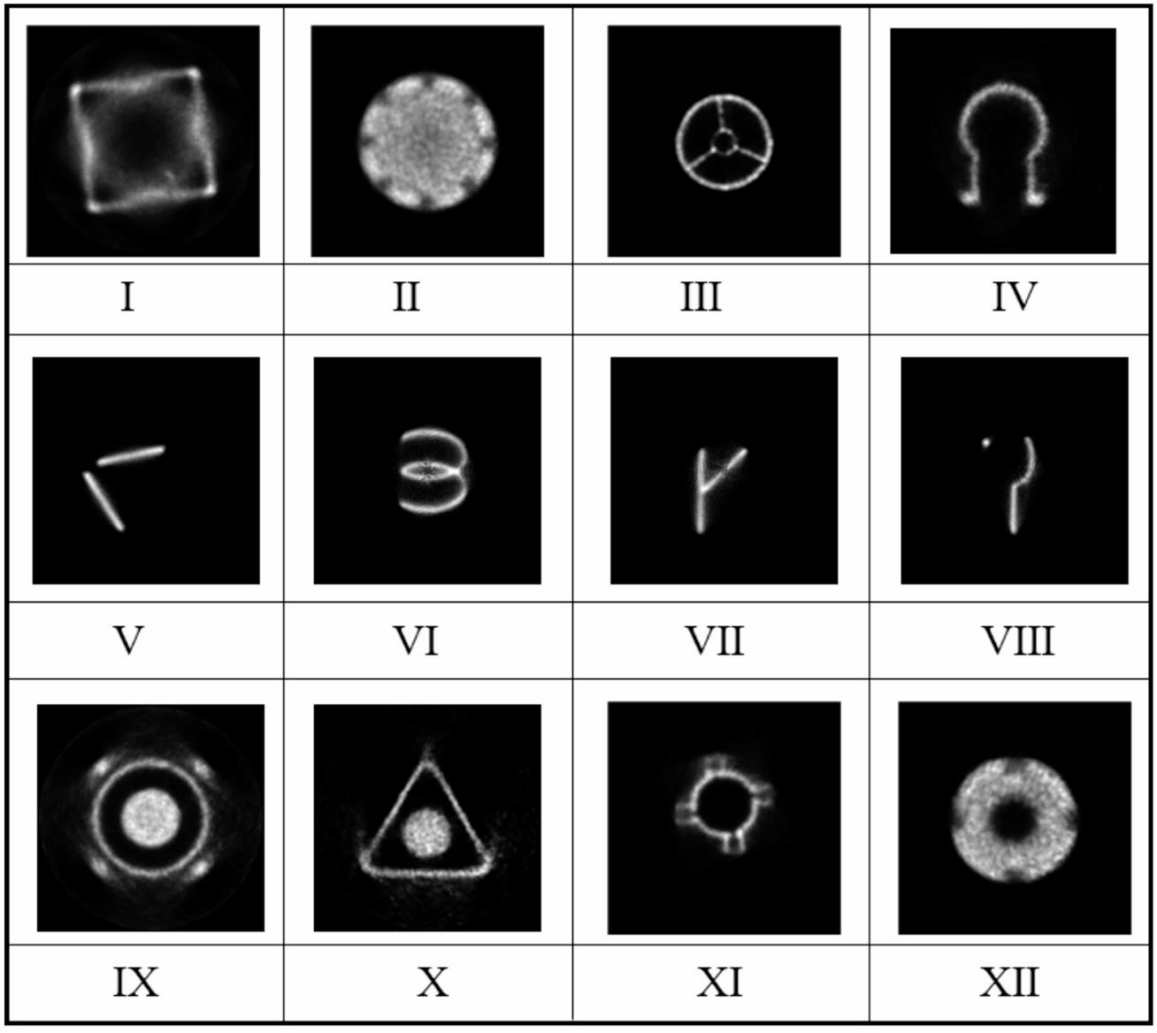
PET image example.

In the simulation, the scanning time was set to 30 s and the nuclide concentration was set to 3000 Bq/cm3. The training set includes 800 PET images, the test data consists of 150 images, and the validation set consists of 50 images. At the same time, the images are down-sampled using bicubic interpolation to the corresponding low- resolution images. Some examples are shown in the Fig. 7.

### Experimental parameters

The experiments were conducted using the PyTorch framework on an NVIDIA 4080Ti GPU. The Adam optimizer was employed with an initial learning rate set to 10 − 4, which was halved after 5000, 10,000 and 20,000 iterations. The momentum parameters were set to $\:{\beta\:}_{1}=0.2,\:{\beta\:}_{2}=0.9$, and the weight decay coefficient $\:\alpha\:=1\times\:{10}^{-5}$.

### Ablation experiment

To evaluate the effectiveness of the texture enhancement module (TEM) and fusion connection module (FCM) in improving the positron-based super-resolution reconstruction model, three ablation study configurations were designed.

N1: TEM removed from the model;

N2: FCM removed from the model;

N3: Original loss function retained without proposed modifications.

**Fig. 8:**
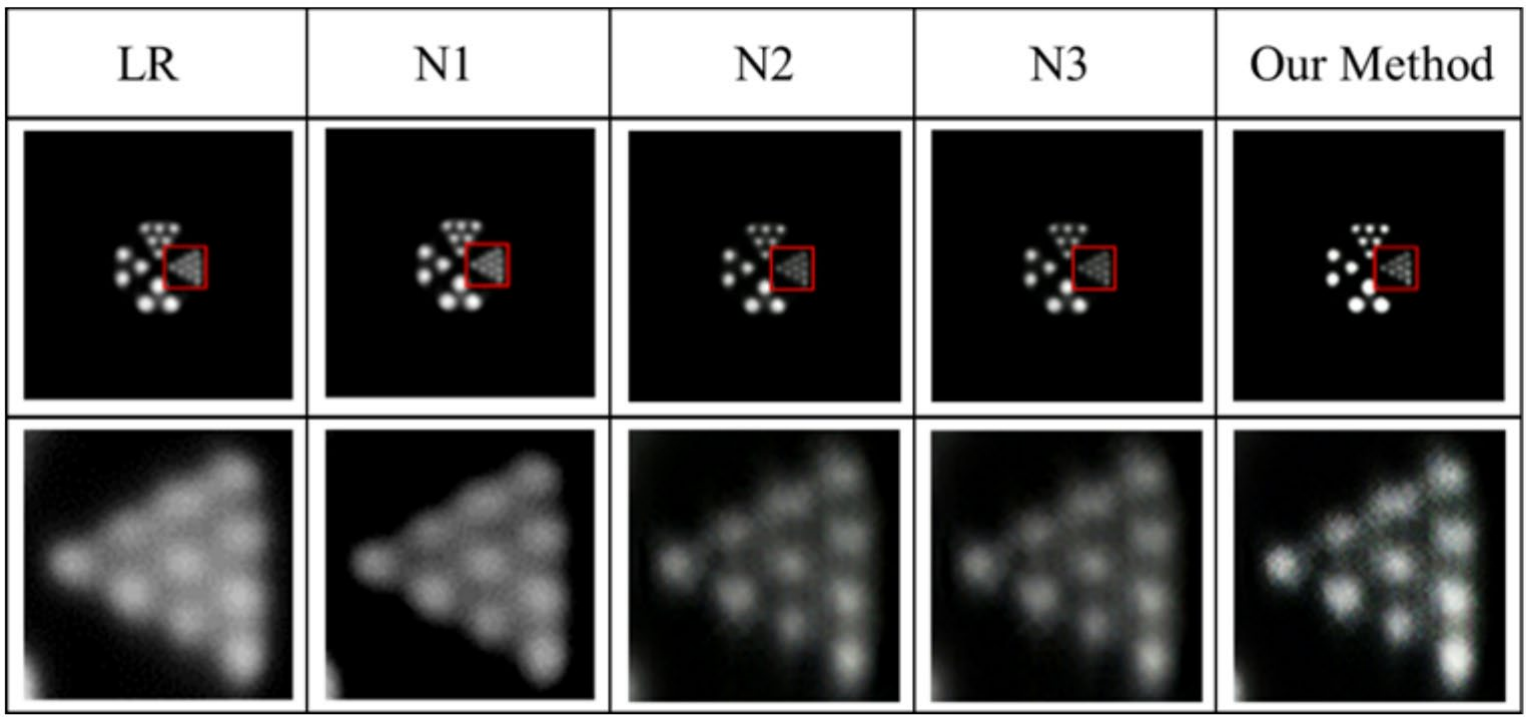
Reconstruction image of ablation experiments.

The experiments were conducted using the same dataset and parameters as those in the proposed method. The experimental results are shown in the Fig. 8, with details highlighted in red boxes and enlarged for better visualization.

From the figure, it can be observed that after removing the texture enhancement module, the reconstruction results appear relatively blurry, and some texture features are lost. After removing the fusion connection module, the reconstructed image lacks certain detailed features, while the sharpening effect becomes more pronounced. The reconstructed image obtained using the original loss function contains some artifacts and lacks sufficient high-frequency information. In contrast, the method proposed in this paper produces the highest-quality super-resolution reconstructed images with a high degree of detail preservation.

The improvement in numerical indicators is clearly visible in the table. Furthermore, PSNR and SSIM were selected as quantitative evaluation metrics, and the corresponding results are presented in Table 1. The improvements in these numerical indicators clearly demonstrate the effectiveness of the proposed method. Therefore, it is essential to construct a texture enhancement network integrated with the super-resolution network and to refine the loss function to achieve superior performance in PET images super-resolution reconstruction.

**Table 1:** Evaluation indicators for ablation experiment images.

Method	PSNR (dB)	SSIM
N1	34.82	0.76
N2	33.26	0.74
N3	35.73	0.83
Our Method	37.49	0.87

### Comparative experiment

**Fig. 9:**
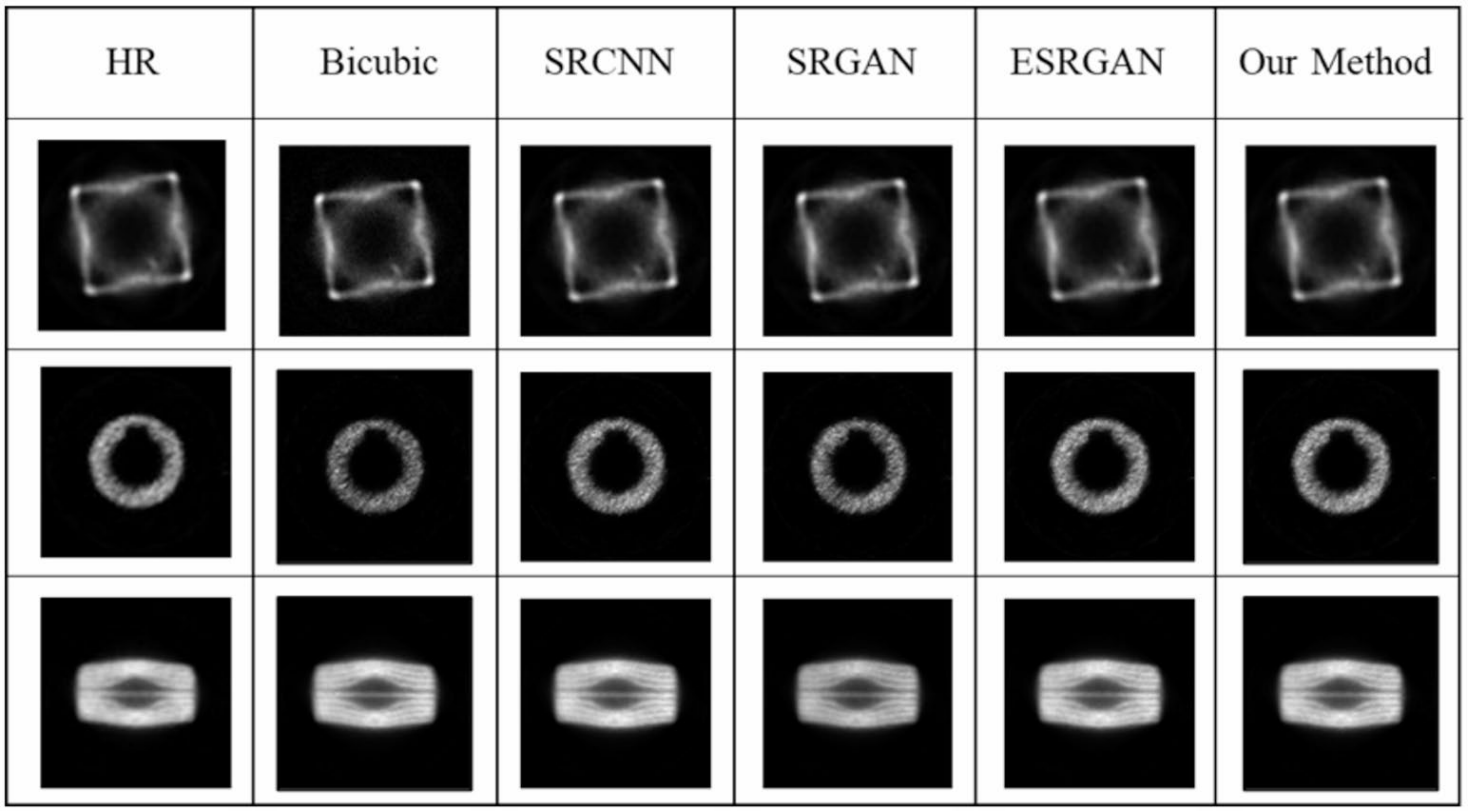
Super-resolution reconstruction of images using different comparison algorithms.

To verify the performance of the proposed PET images super-resolution reconstruction model, a comparative analysis was conducted against several state-of-the-art super-resolution models, including Bicubic interpolation, SRCNN, and SRGAN, with the upscaling factor set to 4. The super-resolution reconstruction results obtained using different algorithms are presented in Fig. 9.

From Fig. 9, it can be seen that SRCNN lacks the ability to learn high-frequency information, resulting in reconstructed images with noticeable blurring. Due to its shallow network depth, SRGAN fails to adequately capture high-frequency features from the input images, producing overly smooth reconstructions with blurred edges. ESRGAN improves the network structure and enhances the overall reconstruction quality; however, it still suffers from image distortion. In comparison, the PET image super-resolution reconstruction algorithm proposed in this study generates reconstructed images with superior visual performance and clearer detailed features. To further provide an objective evaluation of the reconstruction results, the PSNR and SSIM values of each model were calculated for comparison.

**Table 2:** Evaluation indicators for ablation experiment images.

Method	PSNR(dB)	SSIM
Bicubic	35.78	0.786
SRCNN	36.39	0.825
SRGAN	37.64	0.847
ESRGAN	38.82	0.876
Our method	39.24	0.892

As shown in Table 2, the algorithm proposed in this study demonstrates superior image quality, as evidenced by improvements in both PSNR and SSIM compared to other methods.

### Model analysis

A complexity analysis was performed to evaluate the running time and model size of the proposed network. Due to the introduction of a substantial number of residual blocks during the model construction, the number of parameters and the computational complexity of the network increased accordingly. For comparison, SRGAN and ESRGAN — two representative models with comparable reconstruction performance — were selected as benchmarks. The results are presented in Table 3. As shown, although the proposed algorithm exhibits an increase in training time and memory consumption, the difference remains within an acceptable and practical range for real-world applications.

**Table 3:** Evaluation indicators for ablation experiment images.

	SRGAN	ESRGAN	Our method
Training time (h)	4.6	5.4	5.3
Memory size	57.2	58.1	59.5

### Experimental validation

**Fig. 10:**
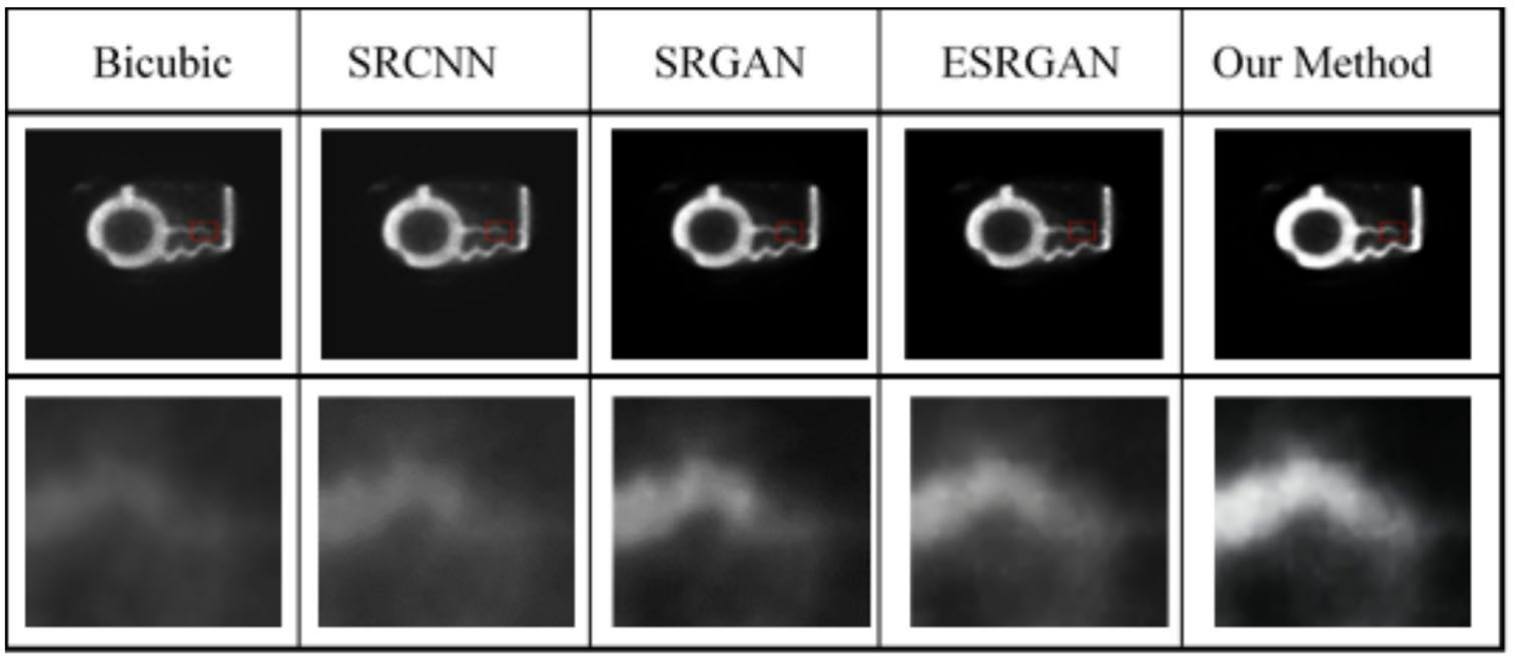
Comparison diagram of super-resolution reconstruction for crack detection.

**Fig. 11:**
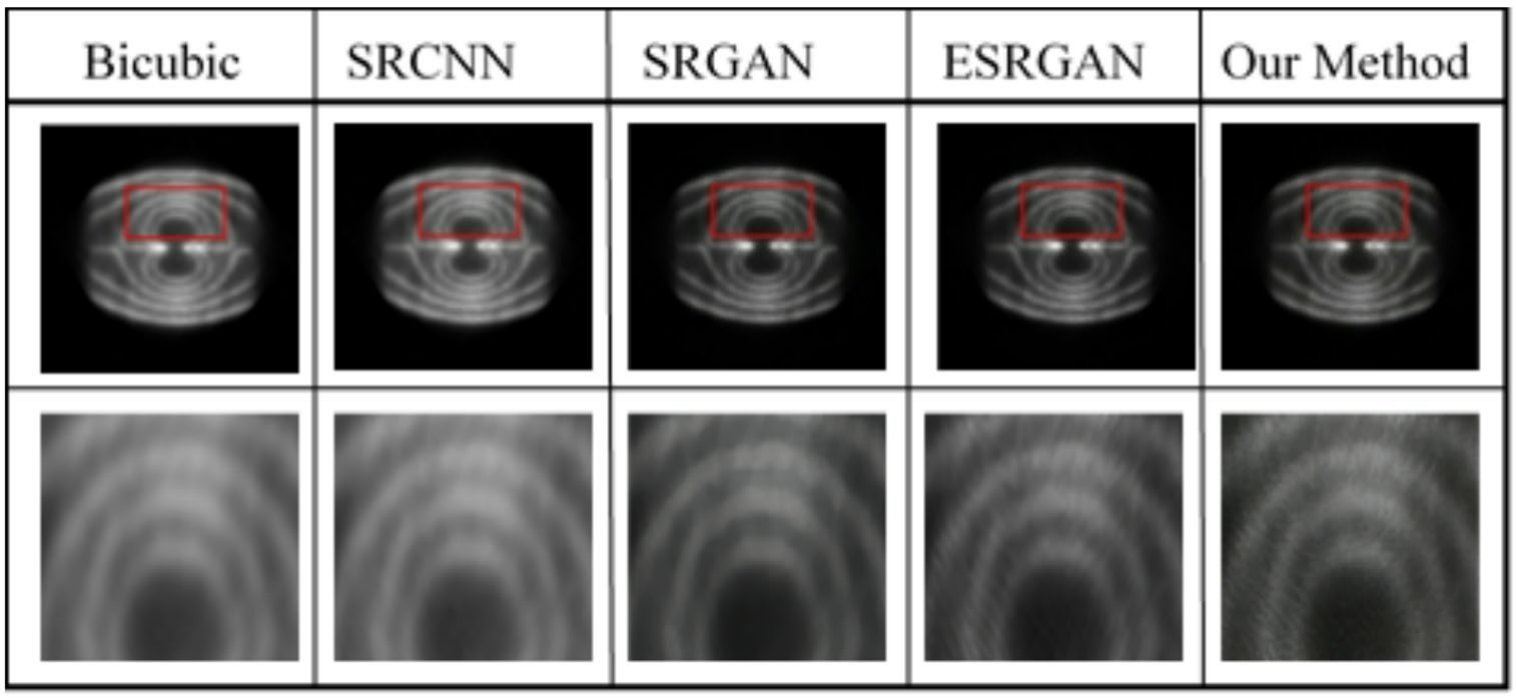
Comparison diagram of super-resolution reconstruction for crack detection.

To verify the practical performance of the algorithm proposed in this study for industrial non-destructive testing scenarios, two sets of on-site experiments were conducted. The first experiment focused on crack detection in industrial components, while the second aimed to monitor the fluid state of hydraulic oil inside an engine. The positron detector employed in these experiments was the Trans-PET Explorist-180, using 18 F as the tracer nuclide with a radioactivity level of approximately 1.85 mCi. The acquisition time for each scan was set to 5 s. The super-resolution reconstruction results are presented in Figs. 10 and 11, where the regions of interest are highlighted with red boxes and enlarged for detailed comparison.

In addition, to objectively evaluate the model performance, and considering the lack of reference standard images in practical applications, Standard Deviation (SD) and Average Gradient (AG) were adopted as objective evaluation metrics. The corresponding results are shown in Table 4.

**Table 4:** Evaluation indicators for ablation experiment images.

	Crack Detection		Flow Field	
	SD	AG	SD	AG
Bicubic	47.75	0.75	45.35	0.72
SRCNN	51.89	0.79	48.57	0.75
SRGAN	63.26	1.04	58.34	0.89
ESRGAN	68.93	1.31	61.57	1.25
Our Method	72.28	2.42	64.36	2.53

It can be clearly observed from the table that the algorithm proposed in this study achieves the highest SD and AG values, indicating superior expression of image details and textures, as well as the highest overall image quality.

## Discussion

The research on positron chromatography imaging technology for industrial nondestructive testing is still in its early stages. However, due to the strong penetration capability of γ photons, it enables internal imaging of confined metal cavities. Compared to surface defect detection methods such as X-rays and ultrasound, this technology holds significant potential for the internal inspection of industrial components. Compared to conventional nondestructive testing methods such as X-ray radiography and ultrasonic inspection, which are also capable of detecting internal structures, positron imaging technology offers unique advantages for visualizing defects within enclosed cavities of industrial components, owing to the strong penetration capability of γ-photons.

The PET images super-resolution reconstruction model proposed in this article integrates prior knowledge and significantly enhances image detail clarity and edge sharpness through the fusion of a texture enhancement network and domain-specific prior knowledge. This approach leads to substantial improvements in PSNR and SSIM metrics.

In addition, simple numerical evaluation metrics alone cannot fully guarantee the quality of the reconstructed images. Unlike natural images, the super-resolution reconstruction performance of industrial PET images must be assessed within the context of their practical application scenarios. The evaluation framework, therefore, requires not only reliable numerical indicators but also convincing visual quality. Moreover, expert judgment from domain professionals is essential to assess the reconstructed images, thereby enhancing the interpretability and application relevance of the reconstruction results.

We also acknowledge several potential limitations of the proposed method. Although the model demonstrates robust performance on both simulated and real datasets, it may be partially influenced by the characteristics of the simulation data, which could lead to overfitting under certain conditions. Additionally, the method may be sensitive to different types and levels of noise in real industrial environments, such as low photon counts, variations in material density, or complex internal cavity geometries, which can affect reconstruction accuracy.

## Conclusions

In response to the challenges of low image resolution and inaccurate texture details produced by positron imaging technology in the field of industrial non-destructive testing, this paper proposes a super-resolution reconstruction method for PET images based on a generative adversarial network that integrates prior knowledge. A texture enhancement network is designed to extract detailed features from the images, and image details are progressively restored by fusing this network with the super-resolution reconstruction network. Meanwhile, the loss function is optimized by incorporating perceptual loss and texture loss, further enhancing the reconstruction performance of the model. Comparative experiments with state-of-the-art super-resolution algorithms demonstrate the superiority of the proposed method in both simulation and practical industrial testing scenarios. Future research will focus on expanding the training dataset, evaluating the generalizability of the proposed method across diverse industrial applications, and promoting its deployment in real-world non-destructive testing environments.

## Data Availability

The data used in the study comes from two parts: the simulation data comes from GATE and it is a special simulation software for PET/SPECT equipment based on Mento Carlo; the real data comes from cooperative enterprises.The datasets used and/or analysed during the current study available from the corresponding author on reasonable request.
